# An Investigation of a New Parameter Based on the Plastic Strain Gradient to Characterize Composite Constraint around the Crack Front at a Low Temperature

**DOI:** 10.3390/ma15030881

**Published:** 2022-01-24

**Authors:** Lingyan Zhao, Zheren Shi, Zheng Wang, Fuqiang Yang

**Affiliations:** 1 School of Science, Xi’an University of Science and Technology, Xi’an 710054, China; yang_afreet@163.com; 2 School of Mechanical Engineering, Xi’an University of Science and Technology, Xi’an 710054, China; szr410826363@163.com (Z.S.); 19205201083@stu.xust.edu.cn (Z.W.)

**Keywords:** stress corrosion cracking, quantitative prediction, composite constraint, fracture toughness, strain gradient

## Abstract

Stress corrosion cracking (SCC) is an important destruction form of materials such as stainless steel, nickel-based alloy and their welded components in nuclear reactor pressure vessels and pipes. The existing popular quantitative prediction models of SCC crack growth rate are mainly influenced by fracture toughness values *K*_Jc_ or *J*_c_. In particular, the composite constraint, containing the in-plane constraints and out-of-plane constraints around the crack front, has a significant influence on the fracture toughness of structures in nuclear power plants. Since the plastic strain gradient is a characterization parameter of the quantitative prediction model for crack growth rate, it may be a characterization parameter of composite constraint. On the basis of the experimental data at a low temperature of alloy steel 22NiMoCr3-7 used in nuclear pressure vessels, the gradient of equivalent plastic strain *D*_PEEQ_ around the crack fronts at different constraint levels was calculated using the finite element method, which introduces a new non-dimensional constraint parameter *D*_p_, to uniformly characterize the in-plane and out-of-plane constraint effects. Compared with constraint parameters *A*_PEEQ_ or *A*_p_, the process of obtaining parameters *D*_PEEQ_ or *D*_p_ is much simpler and easier. In a wide range, a single correlation curve was drawn between parameter *D*_p_ and normalized fracture toughness values *K*_Jc_/*K*_ref_ or *J*_c_/*J*_ref_ of specimens at a low or high constraint level. Therefore, regardless of whether the constraint levels of the structures or standard specimens are low or high, constraint parameter *D*_p_ can be used to measure their fracture toughness. To build an evaluation method that has structural integrity and safety while containing the composite constraint effects, in addition to accurate theoretical interpretation, further verification experiments, numerical simulations and detailed discussions are still needed.

## 1. Introduction

Stress corrosion cracking (SCC), which often occurs in nuclear powers‘ structural materials in high-temperature and high-pressure water environments, is a failure mode caused by a variety of factors, including tensile stress, susceptible materials and environmental parameters [1,2]. From the microscopic point of view, there are transgranular cracks and intergranular cracks in the SCC region, mainly composed of corrosion defects, such as point corrosion, intergranular corrosion, crevice corrosion, and overall corrosion. As an important form of deterioration of many welded components made of stainless steel and nickel-based alloy, SCC has received increasing attention [3,4]. The existing, popular quantitative prediction model of SCC crack growth rate is mainly influenced by fracture toughness values *K*_Jc_ or *J*_c_. A large number of experiments and theoretical analyses show that constraining factors regarding the crack tip have significant effects on the fracture toughness of structures. 

Due to the ability to resist plastic deformation around the crack front, the composite constraint, which can be divided into in-plane constraints and out-of-plane constraints, has been widely known to be considerably related to the study of material fracture behavior. For example, the measured fracture toughness *K_J_*_c_ is greatly influenced by crack-tip constraint effects [5,6]. The in-plane constraint is mainly related to the specimen or structure size in the direction of crack propagation, such as the uncracked ligament length. Additionally, the out-of-plane constraint is significantly related to the specimen or structure size in the direction parallel to the crack front, for example, the specimen thickness. To improve the accuracy of structural integrity assessment, researchers have proposed many cracking characteristic parameters to determine the driving forces of the cracking behavior in recent decades. The parameters *T*, *A*_2_, *Q*, and *h* are only considered to describe the in-plane constraint effect, and they have no ability to uniformly characterize the composite constraint effect. Therefore, these parameters can be only quantified as one single parameter, respectively. For two-parameter concepts such as *K*-*T* [2], *J*-*T*_stress_ [7,8,9], *J*-*A*_2_ [10], *J*-*Q* [11], and *J-h* [12], with an examination of experimental data, it is found that these investigated concepts differ significantly, and they are not suitable parameters to determine both in-plane and out-of-plane constraints [13]. Constraint parameter *A*_p_ [14,15] is put forward based on the areas that are surrounded by the equivalent plastic strain isolines ahead of crack tips. Additionally, this parameter is able to characterize both of the two types of constraints properly. Meanwhile, the correlation lines of normalized fracture toughness values *K_J_*_c_/*K*_ref_ and *J*_c_/*J*_ref_ with *A*_PEEQ_, *A*_p_ [16] are also obtained. However, the precise calculations of parameters *A*_PEEQ_ and *A*_p_ in the specific areas are very complex and inconvenient, and calculation results are likely affected by grid partition.

To identify a type of composite constraint parameter that can be simply calculated, the constraint parameter should be sensitive to both in-plane and out-of-plane constraints. The slip/dissolution–oxidation model has been considered a reasonable model for the description of SCC behavior in an oxygenated aqueous system in recent decades [17,18,19,20]. The SCC behavior can induce intergranular and transgranular cracks, which will modify the crystal structure of the specimen locally. It should be considered that the grain boundary structure also has a significant effect on the SCC behavior. Based on the strain gradient theory, the strain redistribution and strain rate at the crack tip can be easily obtained at the crack tip [19]. Additionally, the strain rate at the crack tip can lead to interfacial film degradation. According to the elastic–plastic finite element method (FEM), the gradient of the equivalent plastic strain can be calculated [21]. For the conventional electrochemical environment, the crack growth rate is essentially consistent with the plastic strain gradient at the crack tip. Experiments suggest that fracture toughness *K_J_*_c_ is relatively related to the crack growth rate [21] under the composite constraint effect. Since the plastic strain gradient is a characterization parameter of the crack growth rate prediction model, it may be a characterization parameter of composite constraint. This also suggests that the equivalent plastic strain gradient (*D*_PEEQ_) in the fracture plastic zone may be sensitive to composite constraints. Compared with the parameter *A*_PEEQ_ of Yang [14,15], the achievement of parameter *D*_PEEQ_ is much simpler and easier. The calculation process of *D*_PEEQ_ is also very convenient. By means of the reference average gradient of equivalent plastic strain (*D*_ref_) along the crack front of a three-dimensional tensile specimen, the dimensionless constraint parameter *D*_p_ (*D*_p_ = *D*_PEEQ_/*D*_ref_) is supposedly an effective parameter to measure both in-plane and out-of-plane constraint effects. 

In this paper, based on the experimental data of fracture toughness *K_J_*_c_ at a low temperature [13], the distributions of equivalent plastic strain at the crack fronts of the experimental samples were obtained. The new characterization parameters *D*_PEEQ_ and *D*_p_ were obtained by a three-dimensional FEM, and the results show that plastic strain gradient is a combination of composite constraints in a wide range. Whether parameters *D*_PEEQ_ and *D*_p_ can effectively describe and evaluate the composite constraint effect was also investigated. It is helpful to build an evaluation method that has structural integrity and safety while containing the composite constraint effect.

## 2. Theory and Methods

The film slip/dissolution–oxidation often occurs in nuclear-grade steels in high-temperature and oxygen-containing environments. The Ford model [22] is widely accepted to estimate the SCC crack growth rate of nuclear-grade steels structures, in which the crack growth rate d*a*/d*t* can be obtained by

(1) $$\frac{da}{dt}=\kappa_{a}\cdot {\left({\dot{\varepsilon }}_{\mathrm{ct}}\right)}^{m}$$

where ${\dot{\varepsilon }}_{\mathrm{ct}}$ is the strain rate at a specific position ahead of the crack tip, which is represented by d*a/*d*t*. *m* is the exponent of the measured current decay curve. $\kappa_{a}$ represents the oxidation rate constant, which is related to the material properties and electrochemical environment at the crack tip, and it is shown as

(2) $$\kappa_{a}=\frac{M_{\mathrm{mol}}}{\rho \cdot F\cdot z_{c}}\cdot \frac{i_{0}}{1-m}\cdot {\left(\frac{t_{0}}{\varepsilon_{f}}\right)}^{m}$$

where the molecular weight of the metal is represented by *M*_mol_; *ρ* represents its density; *F* represents the Faraday constant; *z*_c_ represents variation in charge because of the oxidation process; $t_{0}$ represents the time before current decay; $i_{0}$ represents the oxidation current density; the degradation strain of oxide film is represented by $\varepsilon_{f}$.

Considering the difficulty to obtain the accurate strain rate at the crack tip, the equivalent plastic strain *ε*_p_ at a characteristic distance *r*_0_ is put forward to substitute *ε*_ct_ in Equation (1), which is

(3) $$\varepsilon_{\mathrm{ct}}=\varepsilon_{p}|r=r_{0}$$

(4) $$\frac{d\varepsilon_{\mathrm{ct}}}{dt}=\frac{d\varepsilon_{p}}{dt}=\frac{d\varepsilon_{p}}{da}\cdot \frac{da}{dt}$$

In Equation (4), the strain rate at *r*_0_ is represented by d*ε*_p_/d*a*. Therefore, the SCC growth rate can be written as

(5) $$\frac{da}{dt}={\left(\kappa_{a}\right)}^{\frac{1}{1-m}}\cdot {\left(\frac{d\varepsilon_{p}}{da}\right)}^{\frac{m}{1-m}}$$

The strain gradient can be written in Equation (6) as follows:

(6) $$\frac{d\varepsilon_{p}}{da}=\frac{\partial \varepsilon_{p}}{\partial a}-\frac{\partial \varepsilon_{p}}{\partial r}$$

Without regard to the first term of Equation (6), d*ε*_p_/d*a* can be expressed as

(7) $$\frac{d\varepsilon_{p}}{da}=-\frac{\partial \varepsilon_{p}}{\partial r}=-\frac{d\varepsilon_{p}}{dr}$$

Finally, the SCC growth rate can be written as

(8) $$\frac{da}{dt}={\left(\kappa_{a}\right)}^{\frac{1}{1-m}}\cdot {\left(\frac{d\varepsilon_{p}}{dr}\right)}^{\frac{m}{1-m}}$$

Equation (8) can be used to calculate the SCC propagation at the defects of actual light water reactor components [21]. The equivalent plastic strain gradient (d*ε*_p_/d*r*) at *r*_0_, which is the only mechanical factor affecting the stress corrosion cracking behavior, can be easily calculated. According to the basic estimating formula, the equivalent plastic strain gradient (d*ε*_p_/d*r*) is considered as the main crack driving force in the stress corrosion cracking propagation process at defects of actual LWR components [23,24,25]. 

A new characterization parameter *D*_PEEQ_ at *r*_0_ was defined as follows:

(9) $$D_{\mathrm{PEEQ}}=\frac{d\varepsilon_{p}}{dr}|r=r_{0}$$

where *D*_PEEQ_ is the average gradient of equivalent plastic strain, and *r*_0_ is a characteristic distance along the crack tip. Additionally, *D*_PEEQ_ can also be written as the average rate of equivalent plastic strain. The characteristic distance *r*_0_ is determined by the contour *rσ*_0_/*J*_ref_ = 2 at the midpoint of the crack front. Here, *J*_ref_ refers to the average value of calculated J-integrals for a reference standard specimen. Along the crack front of the evaluated specimen, *D*_PEEQ_ can be defined as d*ε*_p_/d*r*. In the finite element calculation, at each node in the thickness direction, the corresponding values of *D*_PEEQ_ at *r*_0_ can be calculated by the derivation method and averaging method based on the values of equivalent plastic strain *ε*_p_.

Furthermore, along the crack front of the reference standard specimen at a high restraint level, by means of the equivalent plastic strain gradient (*D*_PEEQ_) and the reference average gradient of equivalent plastic strain (*D*_ref_), a new normalized characterization parameter *D*_p_ was defined as

(10) $$D_{p}=\frac{D_{\mathrm{PEEQ}}}{D_{\mathrm{ref}}}$$

where *D*_ref_ is the average gradient of equivalent plastic strain for a reference standard specimen when the calculated *J* = *J*_ref_.

## 3. The Finite Element Model 

### 3.1. Geometry Model

**Figure 1:**
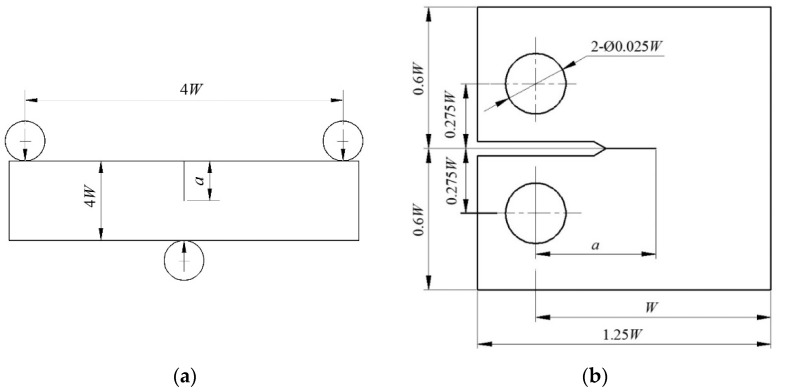
Geometry sizes: (**a**) SE(B); (**b**) C(T) specimens.

According to ASTM Standard E1921-02(2002), the compact tension (C(T)) specimens and three-point bending (SE(B)) specimens are rested at a low temperature of *T*_t_ = −60 °C to represent low and high constraint situations [13]. The geometry and dimension configuration of C(T) and deep notched SE(B)_d_ specimens are shown in Table 1 and Figure 1. The out-of-plane constraint must be considered, which varies with the specimen thickness *B*, while the in-plane constraint changes with variations in the specimen width *W*, the ratio of crack length *a* to *W,* and the specimen type.

### 3.2. Material Properties

The steel 22NiMoCr3-7, which is widely used in the pressure vessel of nuclear power plants, was investigated. The relationship between stress and strain can be written according to [26,27] as follows:

(11) $$\frac{\varepsilon }{\varepsilon_{0}}=\frac{\sigma }{\sigma_{0}}+\alpha {\left(\frac{\sigma }{\sigma_{0}}\right)}^{n}$$

where *ε* is the total of elastic strain and plastic strain; *σ* is the total stress; *ε*_0_ and *σ*_0_ are the material yield strain and yield stress, respectively; *n* represents the material strain hardening exponent; the offset coefficient is represented by *α*. The FEM analysis was carried out by using the mechanical property data of steel 22NiMoCr3-7 at *T*_t_ = −60 °C [13], and the constitutive parameters are given in Table 2.

### 3.3. The Finite Element Model

**Figure 2:**
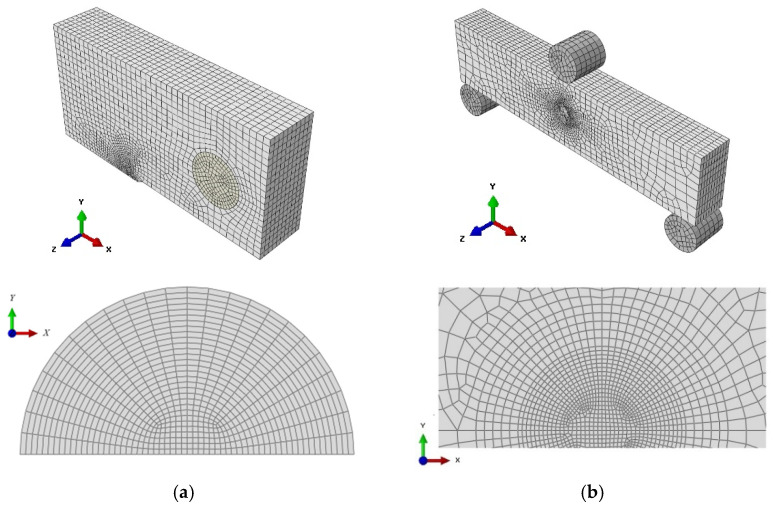
Mesh of global model and mesh around crack front: (**a**) C(T)50 specimen and (**b**) SE(B)10 × 10_d_ specimen.

Three different finite element models were established according to Table 1 in the commercial finite element program ABAQUS. There are two types of C(T) specimens— C(T)25 when the ratio *a/W* = 0.5162 and C(T)50 when the ratio *a/W* = 0.5104, respectively. Additionally, there is one type of SE(B)10 × 10_d_ specimen with the ratio *a/W* = 0.5245. All models used eight-node isoperimetric elements (C3D8R). In order to reduce the calculation time, only 1/4 of the C(T) specimen and 1/2 of the SE(B) specimen were simulated. Additionally, unruptured ligaments were used in symmetrical boundary conditions. The typical meshes (containing 45 280 elements in C(T)50 specimen and 44 047 elements in SE(B)10 × 10_d_ specimen) and refined meshes at crack fronts of these specimens are shown in Figure 2.

## 4. Results

The fracture toughness tests were based on ASTM Standard E1921-02 (2002) at a typical test temperature *T*_t_ = −60 °C. The measured fracture toughness *K_J_*_c_ under a small area yield conditions at the crack fronts was obtained. According to Young’s modulus *E* and Poison’s ratio *ν* in Table 2, the fracture toughness *J*_c_ can be obtained by

(12) $$J_{c}=\frac{1-\nu^{2}}{E}K_{Jc}^{2}$$

To build the relationship between fracture toughness values *K_J_*_c_ and *J*_c_, and parameters *D*_PEEQ_ and *D*_p_ in the simulated experiments, loads were applied to keep the *J* integral at the midpoint of crack front equal to the fracture toughness *J*_c_ listed in Table 3. Then, the stress–strain field around crack fronts was calculated using the commercial finite element code in ABAQUS. 

Experimental data of fracture toughness at *T*_t_ = −60 °C and plastic strain gradient calculated by FEM for constraint analysis are both listed in Table 3.

### 4.1. Equivalent Plastic Strain and its Gradient

**Figure 3:**
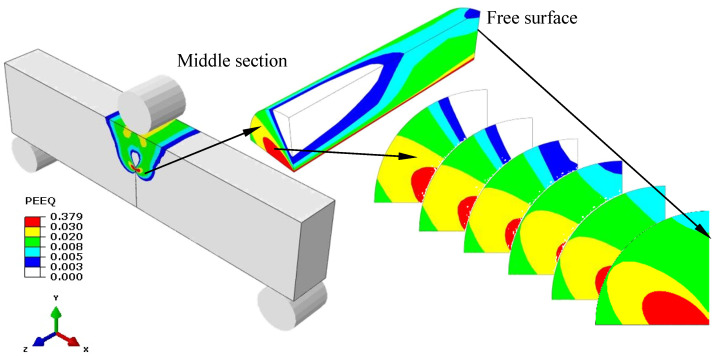
Equivalent plastic strain around crack fronts in sections.

**Figure 4:**
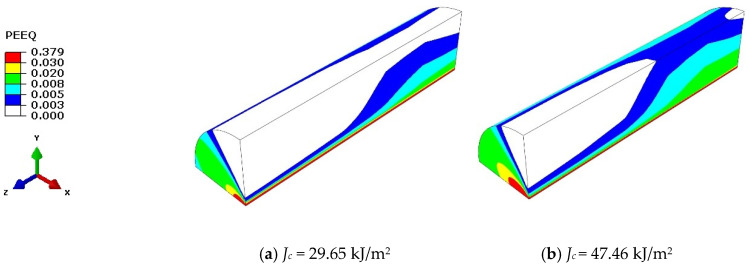

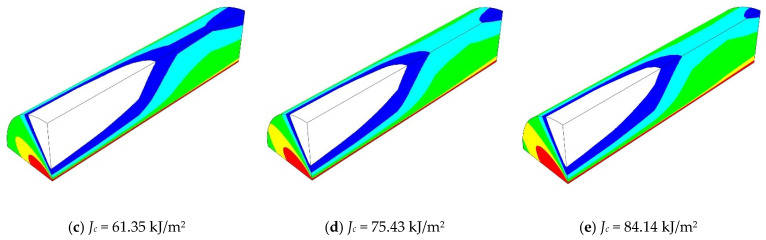
Equivalent plastic strain around crack fronts: (**a**) *J*_c_ = 29.65 kJ/m^2^, (**b**) *J*_c_ = 47.46 kJ/m^2^, (**c**) *J*_c_ = 61.35 kJ/m^2^, (**d**) *J*_c_ = 75.43 kJ/m^2^, and (**e**) *J*_c_ = 84.14 kJ/m^2^.

The distribution of equivalent plastic strain around crack fronts in a SE(B)10 × 10_d_ specimen (*W* = 10 mm, *B* = 10 mm and *a/W* = 0.5245, load *J*_c_ = 84.147 kJ/m^2^) was simulated at *T*_t_ = −60 °C. To acquire a more accurate stress–strain field, a fine mesh was used at the vicinity of the crack front. The specimen thickness was divided into five equal parts. Figure 3 illustrates the strain distribution in six cross-sections along the direction of the specimen thickness. The equivalent plastic strains around the crack fronts of a SE(B)10 × 10_d_ specimen with varying loads *J*_c_ (*J*_c_ = 29.65, 47.46, 61.35, 75.43, and 84.14 kJ/m^2^) are shown in Figure 4. The radius of the study area is 0.5 mm. As the load increases, the equivalent plastic strain around the crack front becomes increasingly higher, and it could be found that a much higher strain area is located on the free surface of the specimen, which implies that the equivalent plastic strain may be a uniform measurement of composite constraint.

**Figure 5:**
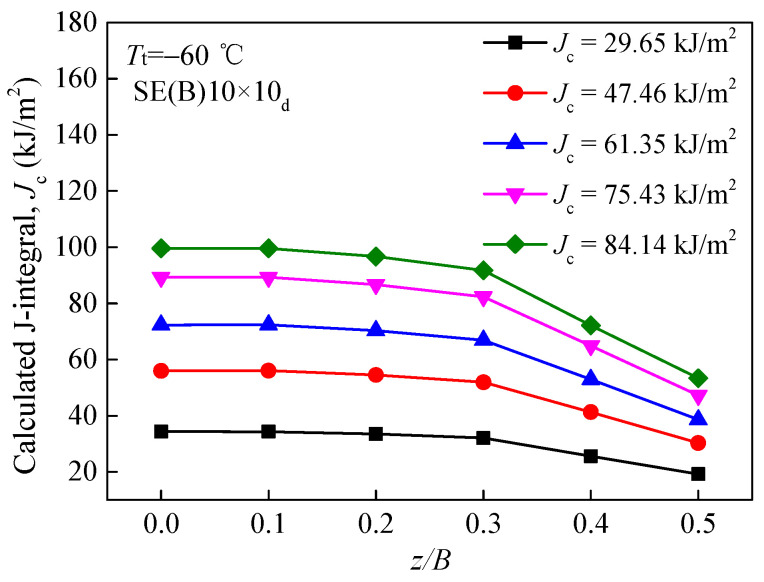
Calculated J integrals around crack fronts.

Figure 5 depicts the curves of calculated J integrals around crack fronts in a SE(B)10 × 10_d_ specimen (*W =* 10 mm, *B* = 10 mm and *a/W* = 0.5245). The *J*_c_ curves were plotted at *T*_t_ = −60℃ against the ratio of *z/B*, where *z/B* = 0 represents the middle section and *z/B* = 0.5 represents the free surface of the specimen. It can be concluded that the central area (*z/B* = 0) has a higher J integral, while the surface area (*z/B* = 0.5) has a lower J integral. Figure 5 also implies that the J integral undergoes a prominent change from the free surface to the middle section of the specimen, indicating that *J*_c_ is not sensitive to the out-of-plane constraint, as it can not effectively characterize the out-of-plane constraint.

**Figure 6:**
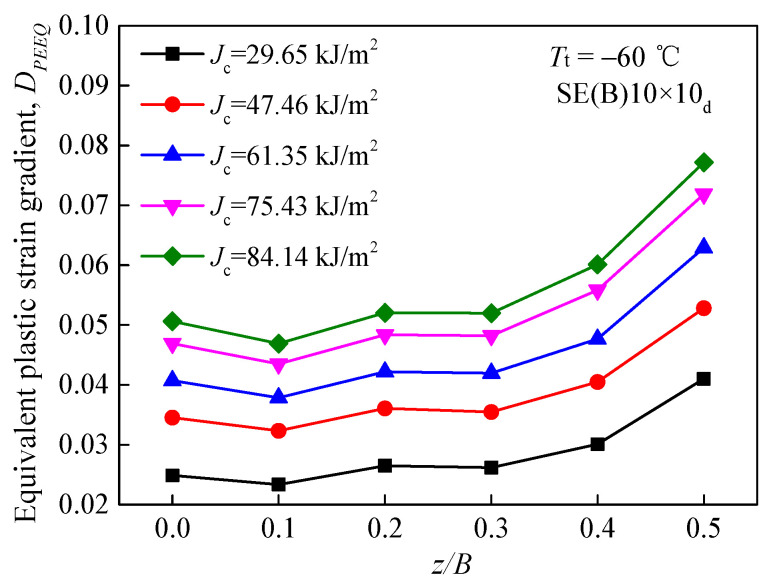
Equivalent plastic strain gradients around crack fronts.

Based on the normalized specimen thickness *z/B* at *T*_t_ = −60 °C, the curves of equivalent plastic strain gradient *D*_PEEQ_ around the crack front in the SE(B)10 × 10_d_ specimen with the increasing *J*_c_ are shown in Figure 6. It is apparent that a lower *D*_PEEQ_ appears in the central area, with *z/B* = 0, and a higher *D*_PEEQ_ appears in the surface area, with *z/B* = 0.5. This indicates that parameter *D*_PEEQ_ is associated with the specimen thickness B, which indicates that *D*_PEEQ_ is sensitive to the out-of-plane constraint. Therefore, *D*_PEEQ_ may be able to characterize it.

### 4.2. Characterization of Composite Constraint

**Figure 7:**
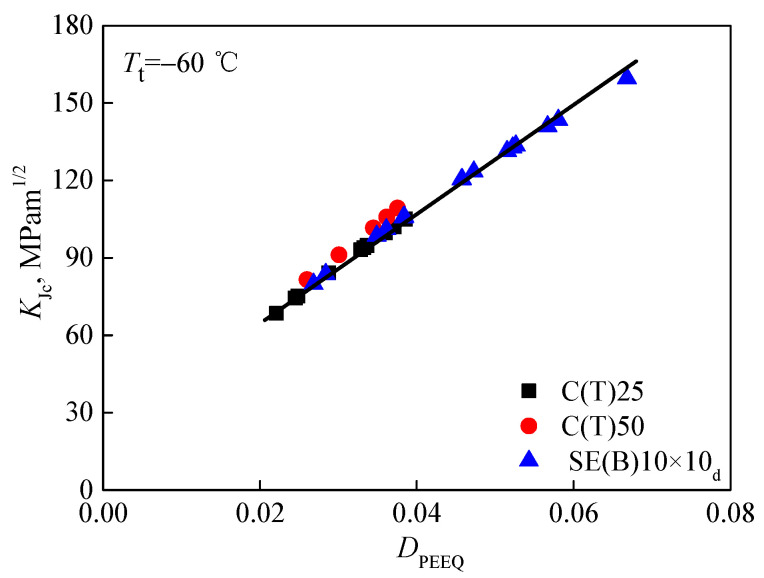
Relation between fracture toughness *K*_Jc_ and constraint parameter *D*_PEEQ_.

**Figure 8:**
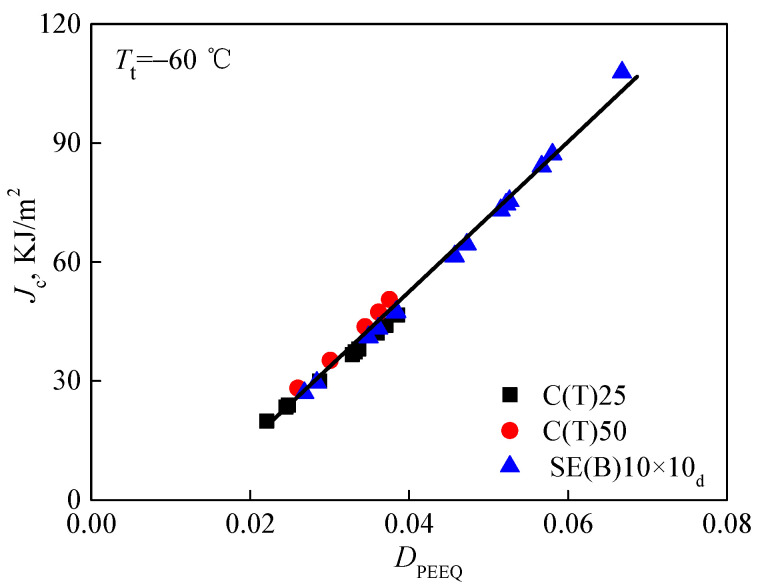
Relation between fracture toughness *J*_c_ and constraint parameter *D*_PEEQ_.

The characteristic distance *r*_0_ is determined by the contour *rσ*_0_/*J*_ref_ = 2 at the midpoint of the crack front. Here, *J*_ref_ refers to the average value of calculated J integrals for a reference standard specimen. Subsequently, when the *J* integral at the midpoint of crack front equals the value of fracture toughness *J*_c_ in Table 3, based on the stress–strain field around crack fronts, the values of the constraint parameter *D*_PEEQ_ in three different specimens, whose in-plane and out-of-plane constraints are totally different, can be calculated. To examine whether the parameter *D*_PEEQ_ can measure the composite constraint, the average values of *D*_PEEQ_ at *r*_0_ through the direction of specimen thickness along the crack front of all specimens were calculated and are also listed in Table 3. Additionally, most particularly, the achievement of *D*_PEEQ_ is much simpler and easier than that of *A*_PEEQ_. Afterward, the fracture toughness values *K_J_*_cc_ and *J*_c_ determined by experiments were drawn as functions of *D*_PEEQ_. In a wide range, the relationships between *K_J_*_c_, *J*_c_, and *D*_PEEQ_ are depicted in Figure 7 and Figure 8. It is obvious that the data are not diffused. The composite constraint parameter *D*_PEEQ_ was found to have a good linear relationship with fracture toughness values *K*_Jc_ and *J*_c_ in a wide range. The values of *K*_Jc_ or *J*_c_ increase with the higher *D*_PEEQ_. *D*_PEEQ_ is apparently sensitive to the normalized fracture toughness values *K*_Jc_ and *J*_c_. The parameter *D*_PEEQ_ is sensitive to both in-plane and out-of-plane constraints.

### 4.3. Correlation of Dp between Composite Constraint

The highly constrained C(T)25 specimen was selected as the standard specimen. The average values of *K*_Jc_ and *J*_c_ of 10 C(T)25 standard specimens were defined as the reference fracture toughness values of *K*_ref_ and *J*_ref_. Along the direction of the specimen thickness, *D*_ref_ was defined as the reference gradient of equivalent plastic strain; it can be obtained from the stress–strain field around the crack front when the *J* integral at the midpoint of crack front equals *J*_ref_. Therefore, the non-dimensional parameter *D*_p_ (*D*_p_ = *D*_PEEQ_/*D*_ref_) could be calculated. The calculated *D*_p_ values are also listed in Table 3.

**Figure 9:**
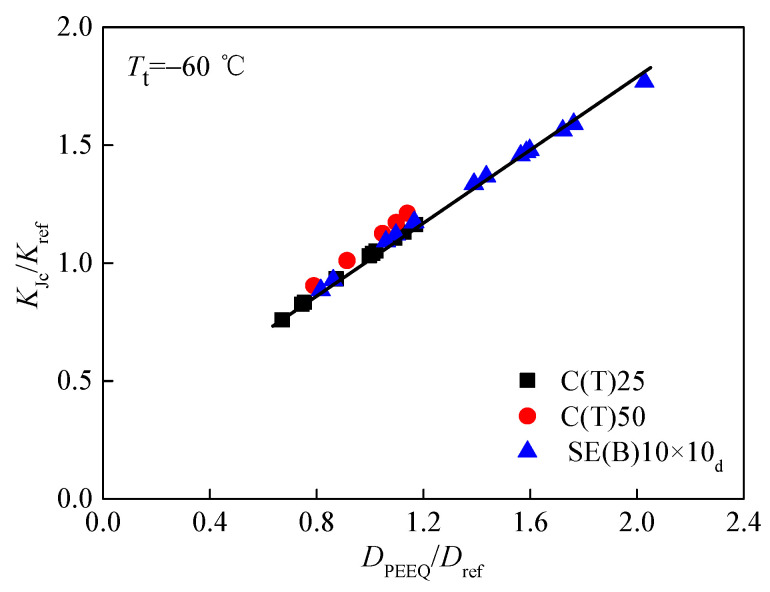
Relation between non-dimensional fracture toughness *K*_Jc_*/K*_ref_ and constraint parameter *D*_p_.

**Figure 10:**
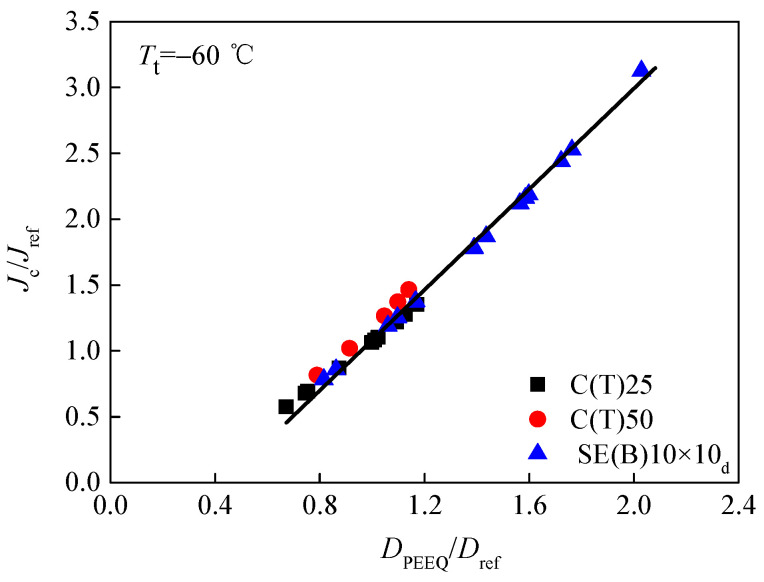
Relation between non-dimensional fracture toughness *J*_c_/*J*_ref_ and constraint parameter *D*_p_.

In Figure 9 and Figure 10, based on the reference fracture toughness values *K*_ref_ and *J*_ref_, and the reference equivalent plastic strain gradient *D*_ref_, the non-dimensional fracture toughness values *K*_Jc_*/K*_ref_ and *J*_c_*/J*_ref_ in a wide range were obtained, and they were used as two functions of the non-dimensional constraint parameter *D*_p_ for all specimens with different composite constraints at the crack fronts. The parameter *D*_p_ (*D*_p_= *D*_PEEQ_/*D*_ref_) also has a uniform relationship with *K*_Jc_/*K*_ref_ and *J*_c_*/J*_ref_ of specimens at low and high constraint levels. Therefore, *D*_p_ can characterize the in-plane constrain, as well as the out-of-plane constrain. The non-dimensional constraint parameter *D*_p_ is able to measure the fracture toughness of different standard specimens or structures at a low or high constraint level.

## 5. Conclusions

Based on the equivalent plastic strain gradient at a characteristic distance *r*_0_ ahead of crack fronts in three types of specimens, constraint parameter *D*_PEEQ_ and non-dimensional constraint parameter *D*_p_ were calculated and analyzed. Different from parameter *A*_PEEQ_, *A*_p_ obtained by Yang [14,15], obtaining parameters *D*_PEEQ_ and *D*_p_ is much simpler and easier. *D*_PEEQ_ and *D*_p_ both have the capability to characterize the composite constraint effect, which was examined in this study. For various specimens at a low or high constraint level, the concept derived from *D*_PEEQ_ and *D*_p_ has a good linear relationship with normalized fracture toughness values *K*_Jc_/*K*_ref_ and *J*_c_*/J*_ref_ in a wide range, and it can be selected as a uniform parameter to measure the composite constraint effect. The correlation between *D*_p_ and *K*_Jc_/*K*_ref_ or *J*_c_*/J*_ref_ can be used to measure the fracture toughness in a wide range. Therefore, regardless of whether the structures or standard specimens have low or high constraint levels, constraint parameter *D*_p_ can be used to measure their fracture toughness. Studies also have shown that the composite constraint at the crack tip has some relationship with the test temperature *T*_t_ [5] and that the correlation curves need to be established between them. To build an evaluation method that has structural integrity and safety while containing the composite constraint effect, in addition to accurate theoretical interpretation, further verification experiments, numerical simulations and detailed discussions are still needed. 

## Figures and Tables

**Table 1:** Specimen sizes [13].

Name	Type	*T*_t_ (°C)	Number	*W* (mm)	*B* (mm)	*a/W*
C(T)25	C(T)	−60	10	50	25	0.5162
C(T)50	C(T)	−60	5	100	50	0.5104
SE(B)10 × 10_d_	SE(B)	−67/−70	15	10	10	0.5245

**Table 2:** Constitutive parameters of 22NiMoCr3-7 steel at −60 °C [13].

Young’s Modulus *E* (MPa)	Poison’s Ratio *ν*	Yield Stress *σ*_0_ (MPa)	Hardening Exponent *n*	Offset Coefficient *α*
215 000	0.3	517	3.7	6.2

**Table 3:** Experimental data of fracture toughness [13] and plastic strain gradient calculated by FEM.

Specimen Types	*K_J_*_c_ (MPa m^1/2^)	*J*_c_ (kJ/m^2^)	*D*_PEEQ_	*D*_p_
C(T)25	68.5	19.86022	0.02210	0.67100
	74.4	23.42873	0.02451	0.74417
	75.2	23.93529	0.02484	0.75419
	84.2	30.00731	0.02877	0.87351
	93.1	36.68616	0.03287	0.99800
	93.9	37.31935	0.03325	1.00953
	94.8	38.03817	0.03367	1.02229
	99.7	42.07201	0.03601	1.09333
	102	44.03553	0.03711	1.12673
	105	46.66395	0.03856	1.17076
C(T)50	81.6	28.18274	0.02600	0.78941
	91.2	35.20405	0.03009	0.91359
	101.6	43.69084	0.03447	1.04658
	105.8	47.37773	0.03617	1.09819
	109.3	50.56421	0.03755	1.14009
SE(B)10 × 10_d_	79.9	27.02069	0.02685	0.81522
	83.7	29.65199	0.02839	0.86197
	98.5	41.06534	0.03489	1.05963
	101.1	43.26187	0.03612	1.09667
	105.7	47.28821	0.03832	1.16347
	105.9	47.46734	0.03841	1.16620
	120.4	61.35584	0.04571	1.38784
	120.5	61.4578	0.04576	1.38936
	123.4	64.45153	0.04727	1.43521
	131.4	73.07918	0.05151	1.56394
	132.7	74.53234	0.05221	1.58519
	133.5	75.43371	0.05264	1.59825
	141.0	84.14749	0.05669	1.72152
	143.5	87.1579	0.05806	1.76281
	159.6	107.8124	0.06681	2.02848

## Data Availability

The data presented in this study are available on request from the corresponding author.

## Nomenclature

*a*	Crack length
*A*_PEEQ_	Area surrounded by the equivalent plastic strain isoline
*A*_p_	A new constraint parameter
*A*_2_	A constraint parameter
*B*	Specimen thickness
*D*_P_	Normalized parameter of the equivalent plastic strain gradient
*D*_PEEQ_	Equivalent plastic strain gradient
*D*_ref_	Average gradient of equivalent plastic strain at *r*_0_ of a reference standard specimen
*E*	Young’s modulus
*F*	Faraday’s constant
*h*	Stress triaxiality
*J*	J integral
*J*_c_	Average of calculated J integrals
*J*_ref_	Average of calculated J integrals for a reference standard specimen
*K*_Jc_	Measured fracture toughness
*K*_ref_	Fracture toughness of a reference standard specimen
*M*_mol_	Molecular weight of the metal atomic
*m*	Oxidation current decay curve
*N*	Hardening exponent in the Ramberg–Osgood equation
*Q*	A constraint parameter
*r*	Distance along the crack front
*r*_0_	Characteristic distance
*T*_t_	Test temperature
*T*_stress_	Intensity of the crack tip’s stress field
$t_{0}$	Time before the current decay onset
*W*	Specimen width
*z*	Thickness coordinate at the crack front
*z*_c_	Charge change caused by oxidation process
*α*	Offset coefficient of the material
*σ*_0_	Yield stress
d*ε*_p_/d*r*	Equivalent plastic strain gradient at a fixed distance ahead of the crack tip
*ε*_0_	Yield strain
*ε*_f_	Oxide film’s degradation strain
${\dot{\varepsilon }}_{\mathrm{ct}}$	Strain rate at a fixed distance ahead of the crack tip
*ε*_p_	Equivalent plastic strain
$\kappa_{a}$	Constant of oxidation rate
*ρ*	Density of the metal
*ν*	Poisson’s ratio

## References

[1] Meng F.M., Wang J.Q., Han E.H., Shoji T., Ke W. (2011). Microstructure near scratch on alloy 690tt and stress corrosion induced by scratching. Acta Metall. Sin..

[2] Xue H., Ogawa K., Shoji T. (2009). Effect of welded mechanical heterogeneity on local stress and strain ahead of stationary and growing crack tips. Nucl. Eng. Des..

[3] Zhao L.Y., Cui Y.H., Yang F.Q., Xue H. (2018). Analysis on crack driving force at stress corrosion cracking tip induced by scratch in Nickel-based alloy. Rare Met. Mater. Eng..

[4] Horn R.M., Gordon G.M., Ford F.P., Cowan R.L. (1997). Experience and assessment of stress corrosion cracking in L-grade stainless steel BWR internals. Nucl. Eng. Des..

[5] Hohe J., Hebel J., Friedmann V., Siegele D. (2007). Probabilistic failure assessment of ferritic steels using the master curve approach including constraint effects. Eng. Fract. Mech..

[6] Neimitz A. (2006). Fracture toughness of structural elements: The influence of the in-and out-of-plane constraints on fracture toughness. Mater. Sci..

[7] Guo W. (1993). Elastoplastic three dimensional crack border field—II. Asymptotic solution for the field. Eng. Fract. Mech..

[8] Guo W. (1995). Elasto-plastic three-dimensional crack border field-III. Fracture parameters. Eng. Fract. Mech..

[9] She C., Guo W. (2007). The out-of-plane constraint of mixed-mode cracks in thin elastic plates. Int. J. Solids Struct..

[10] Chao Y.J., Yang S., Sutton M.A. (1994). On the fracture of solids characterized by one or two parameters: Theory and practice. J. Mech. Phys. Solids.

[11] O’Dowd N., Shih C. (1991). Family of crack-tip fields characterized by a triaxiality parameter—I. Structure of fields. J. Mech. Phys. Solids.

[12] Clausmeyer H., Kussmaul K., Roos E. (1991). Influence of stress state on the failure behavior of cracked components made of steel. Appl. Mech. Rev..

[13] Hebel J., Hohe J., Friedmann V., Siegele D. (2007). Experimental and numerical analysis of in-plane and out-of-plane crack tip constraint characterization by secondary fracture parameters. Int. J. Fract..

[14] Yang J., Wang G.Z., Xuan F.Z., Yu S.D. (2013). Unified characterisation of in-plane and out-of-plane constraint based on crack-tip equivalent plastic strain. Fatigue Fract. Eng. Mater. Struct..

[15] Yang J., Wang G.Z., Xuan F.Z., Tu S.T. (2014). Unified correlation of in-plane and out-of-plane constraint with fracture resistance of a dissimilar metal welded joint. Eng. Fract. Mech..

[16] Mu M.Y., Wang G.Z., Xuan F.Z., Tu S.T. (2014). Unified parameter of in-plane and out-of-plane constraint effects and its correlation with brittle fracture toughness of steel. Int. J. Fract..

[17] Andresen P.L., Ford F. (1994). Fundamental modeling of environmental cracking for improved design and lifetime evaluation in BWRs. Int. J. Press. Vessel. Pip..

[18] Shoji T., Lu Z., Murakami H. (2010). Formulating stress corrosion cracking growth rates by combination of crack tip mechanics and crack tip oxidation kinetics. Corros. Sci..

[19] Yang F.Q., Xue H., Zhao L.Y., Fang X.R. (2014). A quantitative prediction model of SCC rate for nuclear structure materials in high temperature water based on crack tip creep strain rate. Nucl. Eng. Des..

[20] Zaferani S.H., Miresmaeili R., Pourcharmi M.K. (2017). Mechanistic models for environmentally-assisted cracking in sour service. Eng. Fail. Anal..

[21] Xue H., Shoji T. (2006). Quantitative prediction of EAC crack growth rate of sensitized type 304 stainless steel in boiling water reactor environments based on EPFEM. J. Press. Vessel. Technol..

[22] Ford F.P. (1996). Quantitative prediction of environmentally assisted cracking. Corrosion..

[23] Lu Z., Shoji T., Meng F., Xue H., Qiu Y., Takeda Y., Negishi K. (2011). Characterization of microstructure and local deformation in 316NG weld heat-affected zone and stress corrosion cracking in high temperature water. Corros. Sci..

[24] Peng Q., Kwon J., Shoji T. (2004). Development of a fundamental crack tip strain rate equation and its application to quantitative prediction of stress corrosion cracking of stainless steels in high temperature oxygenated water. J. Nucl. Mater..

[25] Koshiishi M., Hashimoto T., Obata R. (2017). Application of the FRI crack growth model for neutron-irradiated stainless steels in high-temperature water of a boiling water reactor environment. Corros. Sci..

[26] Panter J., Viguier B., Cloué J.-M., Foucault M., Combrade P., Andrieu E. (2006). Influence of oxide films on primary water stress corrosion cracking initiation of alloy 600. J. Nucl. Mater..

[27] Xue H., Wang Z., Wang S., He J., Yang H. (2021). Characterization of mechanical heterogeneity in dissimilar metal welded joints. Mater..

